# Immunopathology of SARS-CoV-2 Infection: A Focus on T Regulatory and B Cell Responses in Children Compared with Adults

**DOI:** 10.3390/children9050681

**Published:** 2022-05-07

**Authors:** Gabriele Di Sante, Danilo Buonsenso, Cristina De Rose, Maria Tredicine, Ivana Palucci, Flavio De Maio, Chiara Camponeschi, Nicola Bonadia, Daniele Biasucci, Davide Pata, Antonio Chiaretti, Piero Valentini, Francesco Ria, Maurizio Sanguinetti, Michela Sali

**Affiliations:** 1 Dipartimento di Medicina e Chirurgia Traslazionale, Sezione di Patologia Generale, Università Cattolica del Sacro Cuore, 00168 Rome, Italy; gabriele.disante@unicatt.it (G.D.S.); mariatredicine@gmail.com (M.T.); chiara.camponeschi94@gmail.com (C.C.); francesco.ria@unicatt.it (F.R.); 2 Dipartimento di Medicina Traslazionale, Sezione di Anatomia Umana, Clinica e Forense, Università degli studi di Perugia, 06123 Perugia, Italy; 3 Dipartimento di Scienze di Laboratorio e Infettivologiche, Fondazione Policlinico Universitario A. Gemelli IRCCS, 00168 Rome, Italy; ivana.palucci@unicatt.it (I.P.); demaioflavio@yahoo.it (F.D.M.); maurizio.sanguinetti@unicatt.it (M.S.); michela.sali@unicatt.it (M.S.); 4 Dipartimento della Salute della Donna e del Bambino e di Sanità, Fondazione Policlinico Universitario A. Gemelli IRCCS, 00168 Rome, Italy; cristyderose@gmail.com (C.D.R.); davide.pata01@gmail.com (D.P.); antonio.chiaretti@policlinicogemelli.it (A.C.); piero.valentini@policlinicogemelli.it (P.V.); 5 Global Health Research Center, Università Cattolica del Sacro Cuore, 00168 Roma, Italy; 6 Dipartimento di Scienze Biotecnologiche di Base, Cliniche Intensivologiche e Perioperatorie—Sezione di Microbiologia, Università Cattolica del S. Cuore, 00168 Rome, Italy; 7 Dipartimento di Medicina di Emergenza, Fondazione Policlinico Universitario A. Gemelli IRCCS, 00168 Rome, Italy; nicola.bonadia@policlinicogemelli.it; 8 Dipartimento di Anestesia e Terapia Intensiva, Fondazione Policlinico Universitario A. Gemelli IRCCS, 00168 Rome, Italy; daniele.biasucci@policlinicogemelli.it

**Keywords:** childhood SARS-CoV-2 infection, COVID-19 immunopathology, T cells

## Abstract

While the clinical impact of COVID-19 on adults has been massive, the majority of children develop pauci-symptomatic or even asymptomatic infection and only a minority of the latter develop a fatal outcome. The reasons of such differences are not yet established. We examined cytokines in sera and Th and B cell subpopulations in peripheral blood mononuclear cells (PBMC) from 40 children (<18 years old), evaluating the impact of COVID-19 infection during the pandemic’s first waves. We correlated our results with clinical symptoms and compared them to samples obtained from 16 infected adults and 7 healthy controls. While IL6 levels were lower in SARS-CoV-2^+^ children as compared to adult patients, the expression of other pro-inflammatory cytokines such as IFNγ and TNFα directly correlated with early age infection and symptoms. Th and B cell subsets were modified during pediatric infection differently with respect to adult patients and controls and within the pediatric group based on age. Low levels of IgD^−^ CD27^+^ memory B cells correlated with absent/mild symptoms. On the contrary, high levels of FoxP3^+^/CD25^high^ T-Regs associated with a moderate–severe clinical course in the childhood. These T and B cells subsets did not associate with severity in infected adults, with children showing a predominant expansion of immature B lymphocytes and natural regulatory T cells. This study shows differences in immunopathology of SARS-CoV-2 infection in children compared with adults. Moreover, these data could provide information that can drive vaccination endpoints for children.

## 1. Introduction

For more than two years now, the world has been dealing with the pandemic generated by SARS-CoV-2 (CoV2) infection, which to date has caused millions of deaths. Since its first description in China, the pathology caused by CoV2 has immediately shown to have a strong clinical impact especially in the most fragile population such as the elderly or those affected by other comorbidities.

While the clinical impact of CoV2 on adults has been massive, children have been relatively spared. Comprehensive reports about the clinical characteristics of CoV2 in children from China, the US [1], and Europe [2] showed that most children develop pauci-symptomatic or even asymptomatic infection. Pediatric cases classified as severe and critical (respectively, 2.5% and 0.6%) are significantly less frequent than in adults [3,4,5,6,7] and, providentially, only a tiny minority of them die [8,9]. Age disparities observed in severe cases might be due to a lower susceptibility of children to infection, a lower propensity to showing clinical symptoms, or both. A mathematical model based on epidemiological data from China, Italy, Japan, Singapore, Canada, and South Korea, estimated that susceptibility to infection in subjects younger than 20 years might be half that of adults, with clinical symptoms manifesting from 21% of infections in 10–19-year-old, to 69% in individuals aged over 70 years [10].

Currently, there is an urgent need to understand better the molecular determinants of protective and pathogenic immune responses in CoV2. Several authors suggested the role of a “cytokine storm” in adults with CoV2 [11]. Conversely, the immunological immune responses in children with CoV2 have not yet been comprehensively addressed. Previous studies suggested a stronger innate immune response in children [12,13]; however, detailed B and T cell profiling of children with CoV2 of different severities has not yet been extensively investigated. Given the importance of B and CD4^+^ T cells in antiviral immunity, studying these adaptive immune cell populations is likely to provide insights into the nature of host responses observed in patients with CoV2. A remarkable heterogeneity has been demonstrated in CoV2 specific CD4^+^ T cell subsets across individual patients and with differing severity of CoV2 [14], possibly related to pre-existing T cell responses to other coronaviruses cross-reactive with CoV2 in some, but not all, individuals [15].

To date, pediatric studies mainly focused on understanding the immunological bases of Multisystem Inflammatory Syndrome of Children (MIS-C) and its differences from acute CoV2 [16,17], while a definition of immunological responses in children with different severity of CoV2 and comparison with adults have not yet been performed.

The aim of this study was to explore and compare the well-known immune responses in adults with the poorly studied immune reactivity of children, both infected during the early phases of the pandemic. Specifically, this manuscript focused on Regulatory T lymphocytes (Treg), circulating B cell subpopulations, and cytokine levels in the serum, aiming to define potential features justifying the differences in clinical outcomes among adults and children.

## 2. Materials and Methods

We performed analyses of whole blood, plasma, and sera samples collected from 16 adults and 40 children (<18 years old) evaluated at our CoV2 referral center from October 2020 to March 2021 and 7 pediatric controls of healthy children with no known comorbidities and no infectious diseases during the two months before enrollment and had a negative CoV2 PCR test at the time of blood sampling. The healthy control group was recruited by healthcare professionals with healthy children that agreed to volunteer for the study. Samples were collected at the time of the first diagnosis of acute CoV2 or asymptomatic CoV2 infection. From each patient, we collected epidemiological, demographic, clinical, laboratory, and outcome data. Disease severity was classified as asymptomatic, mild, moderate, severe (dyspnea, hypoxia, or more than 50% lung involvement on imaging), or critical (respiratory failure, shock, or multiorgan system dysfunction) according to literature [1,11,18]. For pediatric CoV2 the following classification was used:

- Asymptomatic infection: without any clinical symptoms and signs, nor abnormal radiologic findings.
- Mild: symptoms of acute upper respiratory tract infection, including fever, fatigue, myalgia, cough, sore throat, runny nose, and sneezing.
- Moderate: pneumonia, associated often but not necessarily with fever, and cough (mostly dry cough, followed by productive cough); oxygen saturation is >92% without any hypoxia manifestation.
- Severe: early respiratory symptoms, such as fever and cough, may be accompanied by gastrointestinal symptoms, such as diarrhea. Oxygen saturation is <92% with other hypoxia manifestations.

### 2.1. Inclusion and Exclusion Criteria

Pediatric and adult patients with a microbiologically confirmed diagnosis (based on CoV2 detected on nasopharyngeal swab by RT-PCR) of CoV2 and CoV2^−^ children that gave the consent to store the samples for CoV2 research purposes and signed the consent were included in the analyses. Children were defined as patients younger than 18 years of age. Only patients with symptoms that started less than 7 days before our evaluation were included.

Patients with confirmed or suspected primary or acquired immune compromising conditions, recent or current administration of immune-suppressive therapies, or other diseases affecting the immune system were excluded from the study. Patients that recovered from acute CoV2 that had persistence of viral shedding or chronic symptoms (long COVID-19) were also excluded. Children fulfilling WHO criteria for MIS-C were excluded since recent studies suggest a specific immunological signature for this condition [16,17]. Controls were enrolled among children admitted to the emergency unit who were negative for CoV2 and not diagnosed with other acute infectious diseases as well.

Starting from the literature [19,20] and CDC classification (https://www.cdc.gov/ncbddd/childdevelopment/positiveparenting/index.html, accessed on 18 January 2022) and considering our cohort of children, we distinguished the group comprising newborns, infants, and toddlers (0–3 years) from the group older children, assuming that, due to the maturation of the immune system, the cytokine levels of these two groups could have different profiles (with a progressive increase with age) and types of response to antigen-exposure/activation/maturation.

### 2.2. Cytokines Analyses

The evaluation of the expression levels of different human cytokines (Interferon-γ (IFNγ), Interleukin 6 (IL6), interleukin 1β (IL1β), tumor necrosis factor α (TNFα), interleukin 10 (IL10), interleukin 12p70 (IL12p70), interleukin 2 (IL2) and interleukin 4 (IL4)) in serum collected from patients was performed using the ELLA system (ProteinSimple, San Jose, CA, USA) according to the manufacturer’s protocol.

### 2.3. Flow Cytometry Analysis

To standardize the staining procedures, DURAClone^®^ technology (designed panels of dried and pre-coated antibodies in individual tubes for direct labeling of blood) was used to reduce the number of technical steps and avoid a maximum of biases, as it ensures the exact same quantity of antibodies from the same batch, and it is very stable over time. Whole blood samples were stained using a DURAClone IM Treg Tube and DURAClone IM B cells Tube. Stained cells were tested using the CytoFLEX V5-B5-R3 Flow Cytometer and analyzed with Kaluza Analysis 2.1 software (Beckman Coulter, Pasadena, CA, USA). Examples of gate strategy for Regulatory T (Treg) and B cells analysis are reported in supplementary Figures S1 and S2.

### 2.4. Ethic Committee Approval

The study was approved by the ethic committee of our Institution (ID 3078). Written informed consent was obtained from all participants (in the case of adult cohorts) or legal guardians for patients younger than 18 years of age.

### 2.5. Quantification and Statistical Analysis

Data were analyzed using GraphPad Prism 9.3.1. The statistical details of the experiments are provided in the respective figure legends. Data plotted in linear scale were expressed as Mean + Standard Deviation (SD). Data plotted in logarithmic scales were expressed as Geometric Mean + Geometric Standard Deviation (SD). Two-way ANOVA corrected with Tukey was performed to examine the effects and possible interaction of independent variables. A *p* value ≤ 0.05 was considered significant. Details pertaining to significance were also noted in the respective legends.

To ensure that we were correctly comparing age-unmatched patients we performed linear regression (not shown) of age/percentages of Tregs and B cell subsets finding similar correlations in the groups of adults and children both older and younger than 3 years. Due to the numbers of our cohort, it was not possible to further distinguish between newborns, infants, toddlers, pre-schoolers, early- and middle-children, young teens, and teenagers.

## 3. Results

### 3.1. Study Population

We enrolled 47 children and 16 adults: 40 CoV2 positive children (females = 14, males = 26), 16 adults (females = 3, males = 13), and 7 uninfected children (females = 4, males = 3). Among pediatric patients, 10% were asymptomatic, whereas the remainder showed a moderate/severe (30%) or mild (60%) disease course, as shown in Table 1, in which the clinical features of patients are summarized. Patients’ symptoms started on a mean of 2 days before assessment (min 0, max 5 days). Classification of disease severity in children according to age is reported in the supplementary Figure S3.

### 3.2. Inflammatory Biomarkers in CoV2+ Patients

**Figure 1:**
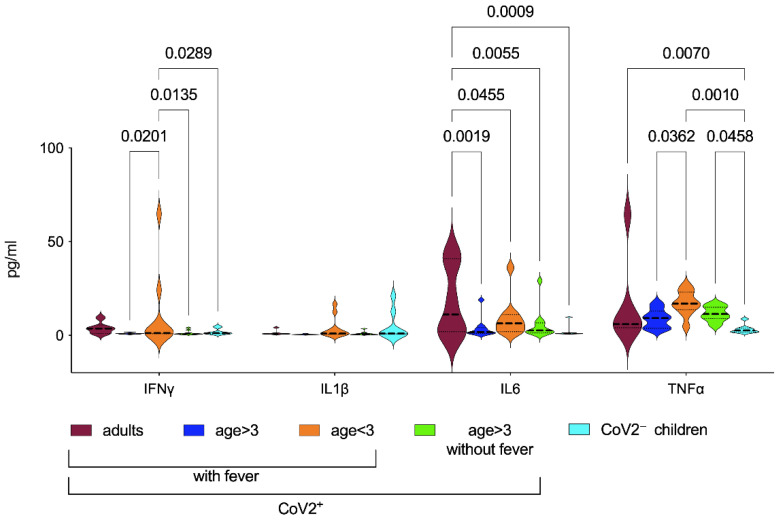
Serum cytokine levels. Cytokine levels were measured in sera samples of patients at data entry, using ELLA Assay (Biotechne, both from R&D Systems). Violin plots compare CoV2 infected groups based on age at onset and the symptom of the fever. Our cohort did not comprise CoV2^+^ adults neither CoV2^+^ children younger than 3 years without fever. Age cut off is described in the method section. Statistical analyses were performed with a two-way ANOVA corrected with Tukey. IFNγ: Interferon-γ, IL6: Interleukin 6, IL1β: interleukin 1 β and TNFα: tumor necrosis factor α.

Innate, Th1, Th2, and immunoregulatory profiles were measured in sera, analyzing IL1β/IL6/TNFα and IFNγ (Table 2 and Figure 1) and IL10, IL12-p70, IL2, and IL4 (Figure S4A) levels at diagnosis of CoV2 infection (early phase) in childhood and compared with infected adults and healthy controls. In order to reduce the heterogeneity of the groups due to different disease severity and symptoms, we subdivided infected children by age (<3 years old comprising newborns, infants, and toddlers vs. >3 years old children, young teens, and teenagers), but also distinguishing the ones with or without fever (expecting a different pattern of cytokines). None of the adult patients were without fever and the control groups comprised children older than 3 years. We observed that although heterogeneously, IFNγ levels were higher in CoV2^+^ children younger than 3 years when compared with the other pediatric groups. IL1β levels were very low in all groups, despite the presence of the fever, and no significant differences were detectable among patients. IL6 levels were higher in CoV2^+^ adults than in all the other pediatric groups; no difference was found among the groups of children for IL6. TNFα levels were significantly higher in CoV2^+^ children younger than 3 years when compared with the other pediatric groups, except when compared with older infected children without fever (Table 2).

We noted that the levels of IFNγ, IL6, and IL1β were directly proportional to age: children older than 3 years displayed significantly increased levels of both cytokines when compared to younger patients (Figure 1); a similar trend was detected also for TNFα. These results supported what has been observed by Pierce et al. [13]. They observed that non-MIS-C had lower concentrations of IL6 and TNFα than the MIS-C group although they did not find a negative correlation with age. They concluded that children with lowest serum concentrations of IL6 and TNFα, cytokines associated with acute respiratory distress syndrome (ARDS), recovered without sequelae. Although IL10 levels were higher than the other cytokines, due to the heterogeneity, it was not possible to find significant differences among groups. A subgroup of infected children with the mild disease showed increased levels of IL10 in sera, undetectable in other patients, and showed a significant correlation between C reactive protein (CRP) and IL10 levels (*p* = 0.02, Figure S4B). Due to the very low levels of IL12p70, IL2, and IL4 in all groups, significant differences were not even detectable among patients.

### 3.3. Peripheral Blood Distribution of B Cells and Treg Subsets in Patients with CoV2 and Controls at Baseline

Peripheral blood samples were tested at diagnosis and compared for B and T regulatory subpopulations. The gating strategies are described in Figures S1 and S2. No significant differences were found in WBC count between pediatric and adult infected patients in peripheral blood. As expected, total amount of lymphocytes (Table 1), both B and T, were higher in children than in adult samples: the proportions were 10.5% ± 7.0% in children versus 3.3% ± 2.1% in adults for B lymphocytes and 23.7% ± 9.1% versus 15.4% ± 10.5% for T cells.

To normalize these age-dependent differences, the percentages of B and T regs subpopulations were gated on total amounts of CD19^+^/CD45^+^ and CD4^+^/CD45^+^, respectively. This gating strategy allowed to reduce not only the bias deriving from the higher number of B and T lymphocytes during childhood, but also within a group of children of similar age to minimize the physiological individual variations.

**Figure 2:**
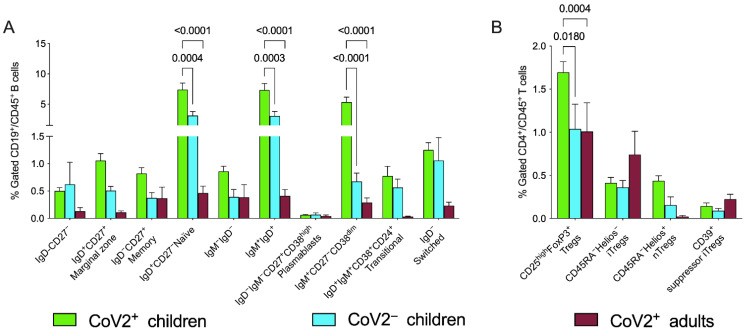
Peripheral blood B and Tregs subsets. Total blood was stained with two different panels of antibodies for B cells (CD45, CD19, CD27, IgD, IGM, CD38, CD24, CD21) and Tregs subsets (CD45, CD3, CD4, CD25, FoxP3, CD39, Helios, and CD45RA). Gating strategy for the identification of cell subsets is described in Figures S1 and S2. (**A**,**B**) Bars show means and SEM of the different distribution of B cell subpopulations and Tregs among groups CoV2-infected children (green bars) and adults (violet bars). Positive and negative markers are displayed with – and + respectively. Highly or moderately expressed markers are displayed as “high” and “dim” respectively.

B cell subpopulations were analyzed taking into consideration the expected numbers/percentages based on the reference ranges for sex and age [21,22] and for children in comparison with the control group. Adults and children showed perturbed distributions of B cell subsets at data entry (Figure 2A); specifically, higher frequencies in the CoV2^+^ children, compared to both CoV2^−^ group and CoV2^+^ adults, were present in IgD^+^CD27^−^ naïve B cells, IgD^+^IgM^+^ and IgM^+^CD27^−^CD38^dim^ B cells as detailed in Figure 2 Statistical significances were determined using two-way ANOVA corrected with Tukey, where each *p* value was adjusted to account for multiple comparisons as shown in Table 3.

We focused our study also on the regulatory compartment of T helper lymphocytes, finding that the CD25^high^FoxP3^+^ Treg subset was significantly expanded in the pediatric group compared to adult patients as well to the control group (Figure 2B and Table 3). Gating strategies with CD39, Helios, and CD45RA (described in methods and displayed in Figure S1) allowed a deep characterization of these subsets, showing trends of predominance of inducible Tregs and mainly suppressor inducible regulatory T cells (iTregs) in CoV2^+^ adults where, as expected, natural Tregs (nTregs) were poorly represented and suggesting a potentially different mechanism of immune regulation in children and adults.

### 3.4. Peripheral Blood Distribution of B Cells and Treg Subsets and Disease Severity

**Figure 3:**
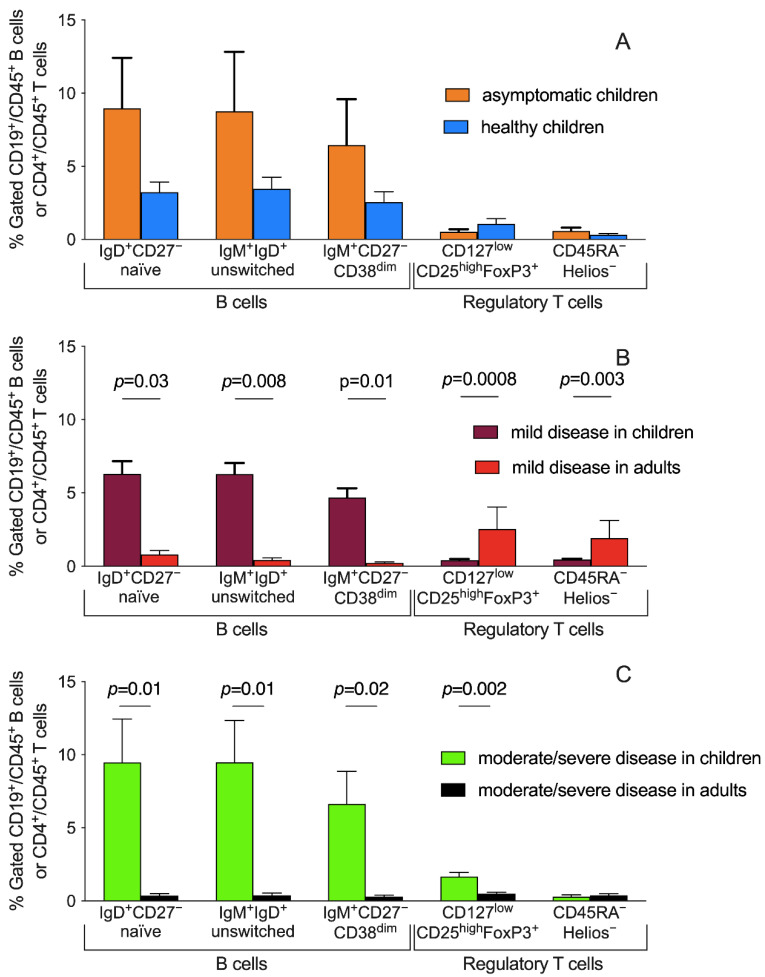
Tregs and B cell subsets and disease severity. Gating strategy for the identification of cell subsets is described in Figures S1 and S2. Bars show means and standard deviations of the different distributions of B cell subpopulations and Tregs. To facilitate the comparison among groups results are displayed based on the disease severity. (**A**) No differences in distributions of displayed B cell and Tregs subsets are detectable comparing asymptomatic CoV2^+^ (*n* = 4, orange bars) with CoV2^−^ (*n* = 7, blue bars) children. (**B**) The graph bars display patients with mild diseases: children (*n* = 24 violet bars) vs. adults (*n* = 3, red bars). (**C**) Patients with a moderate/severe outcome are displayed comparing children (*n* = 12, green bars) and adults (*n* = 13, black bars). Statistical significances were determined using an unpaired *t*-test corrected for multiple comparisons using the Holm–Šidák method. Positive and negative markers are displayed with – and + respectively. Highly or moderately expressed markers are displayed as “high” and “dim” respectively.

Similarly, with cytokine levels, a heterogeneous distribution of B cells and Tregs subsets was detectable among patients based on disease severity. No differences in distributions of B cells and Tregs subsets are detectable comparing asymptomatic CoV2 positive and negative children (Figure 3A). The comparison of patients with mild disease severity (children vs. adults) showed significant differences in IgD^+^CD27^−^ naïve B cells, IgD^+^IgM^+^ unswitched, and IgM^+^CD27^−^CD38^dim^ B cells (*p* = 0.03, *p* = 0.008, and *p* = 0.01, respectively) suggesting a potential role of these subpopulations specifically during mild childhood infection (Figure 3B). Conversely, the T regulatory compartment was consistently expanded more in adults with a mild symptomatology than children (*p* = 0.0008 and *p* = 0.003 for total (CD25^high^FoxP3^+^) and inducible (CD45RA^−^Helios^−^) Tregs, respectively). On the contrary, for patients with a moderate/severe outcome, despite similar significant percentages of B cells described above for patients with mild severity, we observed an opposite distribution of Tregs subsets, higher in children than adults (Figure 3C).

## 4. Discussion

In this study, we provided a detailed immunologic characterization of SARS-CoV-2-infected children, exploring the status of the adaptive immune system, still poorly characterized in the pediatric setting, analyzing the peripheral blood mononuclear cells and sera collected within a mean of 2 days (range 0–5) from symptom onset. To date, studies have found a clear association between the presence of SARS-CoV-2-specific CD4+ T cells and the development of effective adaptive neutralizing immunity has been demonstrated in adults [23,24,25] and children [4,17,26,27], suggesting that rather innate immunity drives the unfavorable clinical course of the disease in adults where it may be responsible for the most severe cases. However, a detailed characterization of B cell subsets and regulatory T cells in adult and pediatric COVID-19 is still missing. In this regard, an important finding of our study is the major expansion of natural Treg in children with COVID-19, compared with healthy children and symptomatic adults.

Initial enthusiasm around the role of the “cytokine storm” (the IL6/IL1β/TNFα pathway in particular) as the main pathophysiological background of CoV2, led to the widespread use of monoclonal antibodies targeting IL6 activity [11]. One of the main difficulties when comparing immune profiles between adults and children and, also, among children of different ages is the bias of the physiological variances in terms of cytokine production and cell number, subsets and maturation. It has been reported that certain cytokine levels are age related and we did not have the possibility to normalize our results for cell percentages [19,20].

Although limited by the low number of patients, our study suggests that CoV2 in children may be characterized by a more complex pattern of cytokine responses, that significantly changes with age. This observation reinforces previous data showing that aggressive inflammatory cascade is unlikely to occur in children. As we show here, in fact, IL6 does not represent a reliable biomarker of disease severity in childhood infection and IL1β was not associated to fever (Figure 1). Recently, Diaz et al. showed that even in patients with MIS-C, IL-6 did not distinguish disease severity, including those that did or did not develop shock [28]. Conversely, Lu et al. found different results [29]. They found that increased IL6 and IL10 in early CoV2 infection in children predicted severe disease. However, the authors also included suspected CoV2 cases, with 75% microbiologically nonconfirmed CoV2 severe cases and 37.5% critical cases. Given the unspecific presentation of CoV2 in children, the misclassification of some cases might also explain the differences compared to what we found in our paper.

We explored the imbalances of the distribution of peripheral B lymphocytes, in line with the distinct immune response/protections of children from the development of severe clinical symptoms. Indeed, we found CoV2-related modifications of several circulating B cell subsets, suggesting that some of these modifications may identify a pediatric signature rather than being associated with disease severity. We were aware that immune profiles during childhood and adulthood are different and similarly also during childhood patients under the age of 3 years have a different distribution of immune cell subsets when compared with older children and adolescents. We reduced this bias through our gating strategy, and we considered the maturation of the immune system, the cytokine production, and the types of response to antigen exposure/activation/maturation (Figure 2A).

The percentage of total memory B cells in CoV2^+^ children decreased according to disease severity. This suggests that memory B cell subsets may play a protective regulatory function potentially interfering with the severity of the disease (Figure 3). This possibility is also supported by the observation that these specific populations were relatively less represented in patients older than 3 years and/or with severe presentations of CoV2 (Figure 3).

As a result of the individuals’ history of immune responses to infections, the dynamics of T lymphocytes in healthy individuals show a reduction in peripheral blood naïve CD4^+^ and CD8^+^ T cell count and the width of the naïve T cell repertoire [30], that increases with age. These differences in cellular frequencies have been associated with lower T cell activation and presence of multifunctional populations in the T cell repertoire upon in vitro stimulation, in children as compared to adults [31]. The reduction in T cell activation and consequently in the T follicular helper response in children was considered one of the possible explanations for milder CoV2 pathogenesis [32].

Furthermore, early-life naïve CD4^+^ T cells tend to differentiate toward a CD25^hi^FoxP3^+^ Treg cell phenotype and persist over an extended period (compared with healthy adult naïve CD4^+^ T cells), leading to an age-related and relatively stable anti-inflammatory profile [33]. The low levels could represent an advantage during pediatric CoV2 and our data suggest an over-representation of the natural Treg compartment compared with adult patients, providing the first evidence that this immune signature may contribute to the differences in disease severity between adults and children.

We showed here that the different distribution of regulatory T subpopulations was not only dependent on disease severity, but also on age: indeed, during adulthood, most of this compartment is composed of almost only inducible Tregs (Figure 2B), while in children natural Tregs could represent an important cohort as it happens in the group with a moderate/severe disease (Figure 3). Our hypothesis that immune-regulatory mechanisms play a pivotal role in the CoV2 infection in childhood is reinforced by the observation that the subgroup of children with a moderate/severe disease course at onset presented a higher concentration of T regs, specifically of natural Tregs, while inducible Tregs seem to play a potential protective role during adulthood, being expanded in the groups of infected adults with mild disease (Figure 3).

Several factors may contribute to a milder severity of CoV2 clinical presentation in children regarding the immunopathogenic response. One possible reason is that the less mature immune system of children is poorly able to elicit cytokine release against viral infection, in contrast with the vigorous immune responses observed in adults [34,35]. Additionally, a stronger innate, trained immune response leads to more effective virus containment/clearance [36]. In support of this theory there is evidence of higher levels of IL17A and IFNγ in children with COV2 in previous studies [13]. Interestingly, despite the huge heterogeneity of our cohort, IFNγ and TNFα levels were significantly higher in the group composed by younger children and with the symptom of fever, suggesting a possible peculiar immune response when CoV2 infection occurs at the early stages of life. Possibly, a Th2-prone milieu could be another possible explanation of mild disease in children, as previously hypothesized in relation to known immune system changes with age [37]. However, another hypothesis not investigated in our study might somehow contribute to a less severe disease in children. Recurrent viral infections that frequently affect children could lead to epigenetic changes in trained immunity making it more effective in clearing CoV2 [38]. Additionally, exposure to commonly circulating HCoV (229E, HKU1, NL63, OC43) might induce pre-existing neutralizing antibodies and T cell immunity to commonly circulating HCoV in younger age groups cross protecting against CoV2 [36], or age-specific distribution of viral receptors might also contribute [39,40,41]. Importantly, endothelial damage and hypercoagulable state with production of thrombi and microclots [42,43], which is more pronounced in adults with comorbidities, can play a role, is not addressed in our study.

Focusing on immune responses, a recent study also characterized immune responses in adults and children, finding that children with CoV2 displayed marked reductions in myeloid cells during infection, most prominent in children under the age of five [44]. However, the authors only included patients with mild disease, limiting the possibility of better understanding the spectrum of immune responses along with different disease severity in children and adults. Similarly, Neeland et al. found that mild CoV2 infection in children is characterized by reduced circulating subsets of monocytes (classical, intermediate, non-classical), dendritic cells, and natural killer cells during the acute phase, with increased proportions of CD63^+^ activated neutrophils [45]. Additionally in this case, however, adults and children with a more severe disease presentation were not included.

A more recent study focused on the characterization of nasal and systemic immune responses in children and adults with CoV2 [46]. The authors found that the systemic response in children was characterized by increases in naive lymphocytes and depletion of natural killer cells, while in adults cytotoxic T cells and interferon-stimulated subpopulations were significantly increased [46]; however, the Treg compartment, which played a role in characterizing disease severity in our cohort, was not specifically investigated. Importantly, Yoshida et al. also analyzed nasal immune responses, showing that the airway epithelium has a higher steady-state expression of IFNγ response genes in children, which may restrict viral spread in children in this cohort [46].

This study has some limitations to address. The main limitation of this study is represented by the low number of children with severe disease enrolled and the overall low number of adult patients. Due to the numbers of our cohort, it was not possible to further distinguish between newborns, infants, toddlers, pre-schoolers, early- and middle-children, young teens, and teenagers. Regarding the healthy controls, this study was completed during the early waves of the pandemic when parents were still highly reluctant to bring children to hospitals and this limited our ability to enroll a larger number of healthy controls. Additionally, we were not able to collect the viral load of our patients. Importantly, this manuscript addresses only part of the complex immune responses that may happen during infection and, specifically, COVID-19. For example, we are aware that Tfh cells play a crucial role for B cell maturation that certainly is a fundamental aspect of the pathogenesis of COVID-19, above all for cases with moderate/high severity. However, we focused on other mechanisms, that in our hypothesis could be crucial in children’s pathogenesis and were still poorly addressed in the literature, specifically the Treg compartment and B cells. In the context of B cell analyses, we did not link these results to antibody quantization or antigen specificity, that were not the aim of this study

However, the more severe spectrum of diseases in children is rare, making a multicentered immunological study necessary to overcome this limitation. Secondly, this study included patients mainly from the first wave of the pandemic and the beginning of the second wave. On one hand, this may have impacted on the decision to admit some mild patients due to the initial uncertainties regarding the impact of CoV2 in children. On the other, although we were not able to obtain sequences of the involved CoV2 variant, this allows us to speculate that this cohort is mainly represented by wild-type virus. This point can also be seen as a potential strength, of our work, since this would provide comparison data for the immune responses in children affected by the wild-type virus.

## 5. Conclusions

In conclusion, our study showed that pediatric CoV2 infection was characterized by a predominant expansion of immature B lymphocytes (IgD^+^CD27^−^ naïve B cells, IgD^+^IgM^+^ unswitched, and IgM^+^CD27^−^CD38^dim^ B cells) and natural regulatory T cells.

Taken together, this study shows a distinct immunopathology of CoV2 infection in childhood, providing information that can explain the low epidemiologic and clinical risk of pediatric subjects. Moreover, these data may prove useful to provide vaccination endpoints for children.

## Figures and Tables

**Table 1:** Study population. Demographic, clinical, and laboratory data of patients are divided into three groups: CoV2^+^ infected and CoV2^−^ children, and infected adults; infected children are subdivided into asymptomatic, with mild and with moderate/severe disease, while adults are separated into affected with mild and moderate/severe disease.

	CoV2^−^ Children (*n* = 7)	CoV2^+^ Children (*n* = 40)			CoV2^+^ Adults (*n* = 16)	
	7	Asympt. 4 (10%)	Mild 24 (60)	Mod./Severe 12 (30)	Mild 3 (18.75)	Mod./Severe 13 (81.25)
Age, mean ± SD, (years)	7.3 ± 5.9	8.3 ± 6.9	8.3 ± 6.9	8.5 ± 4.9	45.4 ± 16.9	64.7 ± 16.6
Female *n*., (%)	4 (57.1)	2 (50)	10 (41.7)	2 (16.7)	1 (33.3)	2 (15.4)
Fever *n*., (%)	N/A	0	15 (62.5)	12 (100)	3 (100)	11 (84.6)
Febrile days, mean ± SD,	0	0	1.8 ± 2.2	2.45 ± 2.66	2 (66.7)	8.5 ± 2.1
Cough, *n*., (%)	0	0	10 (41.7)	8 (66.7)	3 (100)	12 (92.3)
Upper airway symptom, *n*. (%)	0	0	10 (41.7)	3 (25)	2 (66.7)	0
Anosmia, *n*. (%)	0	0	1 (4.2)	1 (8.3)	0	0
Dyspnea, *n*., (%)	0	0	0	1 (8.3)	0	5 (38.5)
Headache, *n*., (%)	0	0	6 (25)	1 (8.3)	0	1 (7.7)
Diarrhea, *n*., (%)	0	0	2 (8.3)	3 (25)	0	0
Lung interstitiopathy, RX, *n*. (%)	0	0	1 (4.2)	6 (50)	0	12 (92.3)
Lung interstitiopathy, Echo, *n*. (%)	0	0	6 (25)	10 (83.3)	Nd	Nd
Hospitalization needed, *n*., (%)	0	0	7 (29.2)	12 (100)	0	8 (61.5)
O2 therapy, *n*., (%)	0	0	0	3 (25)	0	6 (46.2)
HFNC, *n*., (%)	0	0	0	1 (8.3)	0	5 (38.5)
Corticosteroids treat., *n*., (%)	0	0	0	5 (41.7)	0	4 (30.8)
IGIV, *n*., (%)	0	0	0	2 (16.7)	0	0
Antibiotic(s) treat., *n*., (%)	0	0	0	3 (25)	0	3 (23.1)
RBC × 10^12^/L	Nd	4.6 ± 0.6	4.9 ± 0.4	4.7 ± 0.6	3.9 ± 0.3	4.2 ± 1.4
WBC cells × 10^9^/L	Nd	10 ± 4.6	8.7 ± 3.5	10.6 ± 3.4	8.3 ± 1.1	9.1 ± 2.4
Lymphocytes × 10^9^/L	Nd	4.2 ± 2.3	3.3 ± 1.5	4.2 ± 2.9	1.5 ± 0.3	1.8 ± 1.3
Neutrophils × 10^9^/L	Nd	4.7 ± 2.2	4.4 ± 2.8	5.5 ± 2.6	6.1 ± 0.9	6.7 ± 1.5
Monocytes × 10^9^/L	Nd	2.8 ± 4.3	0.6 ± 2.9	0.8 ± 2.8	0.8 ± 0.1	0.9 ± 0.7
CRP, g/L	Nd	39 ± 48.1	73.9 ± 160.4	206.3 ± 240.6	47.9 ± 30.1	69.7 ± 38.3

Abbreviations: HFNC: high flow nasal cannula; IGIV: immune globulin (intravenous); WBC: white blood cells; RBC: red blood cells; CRP: C reactive protein; NA: not available. Nd: not detected.

**Table 2:** Comparison of cytokine expression levels. The data refers to Figure 1 extracted concentrations (mean ± standard deviation, pg/mL) and *p* values were calculated applying two-way ANOVA corrected with Tukey. Comparisons among the different variables (CoV2 positivity, age, and fever) are displayed in the right columns of the table. Significant differences among adults and children are discussed in the results section.

	CoV2^+^				CoV2^−^ Children	*p*-Values					
	With Fever			No Fever		b vs. c	b vs. E	b vs. d	c vs. e	c vs. d	d vs. e
	Adults	Age > 3	Age < 3	Age > 3							
	a	b	c	d	e						
IL1β	1.4 ± 1.4	0.5 ± 0.2	3.9 ± 5.8	0.9 ± 0.9	4.9 ± 7.7	0.38	0.29	0.91	0.82	0.43	0.33
IL6	18.2 ± 19.3	3.8 ± 5.5	9.0 ± 10.2	5.6 ± 8.2	2.1 ± 3.1	0.19	0.68	0.64	0.1	0.38	0.39
TNFα	16.3 ± 23.9	8.9 ± 5.0	17.2 ± 6.4	11.5 ± 4.0	3.3 ± 2.4	0.04	0.18	0.5	0.001	0.14	0.05
IFNγ	3.8 ± 3.1	1.0 ± 0.4	11.4 ± 21.4	1.3 ± 1.2	1.9 ± 1.6	0.02	0.83	0.94	0.03	0.01	0.87

**Table 3:** Comparison of B and Tregs populations. The data refer to Figure 2 extracted percentages (mean ± standard deviation) and *p* values were calculated applying two-way ANOVA corrected with Tukey.

	Children		Adults	*p*-Values	
	CoV2^+^	CoV2^−^	CoV2^+^	a	b
IgD^+^CD27^−^ naïve B cells	7.4 ± 6.4	3.1 ± 1.8	0.5 ± 0.5	0.0004	<0.0001
IgD^+^IgM^+^ B cells	7.3 ± 6.4	3.1 ± 2.0	0.4 ± 0.5	0.0003	<0.0001
IgM^+^CD27^−^CD38^dim^ B cells	5.3 ± 4.9	0.7 ± 0.4	0.3 ± 0.3	<0.0001	<0.0001
CD25^high^FOXP3^+^ Treg	1.7 ± 0.8	1 ± 0.8	1 ± 1.3	0.018	0.0004

a: comparison among CoV2 positive and negative children; b: comparison among CoV2-positive adults and children. CoV2 positive and negative patients are displayed with – and + respectively.

## Data Availability

Data available upon request to the corresponding author.

## Supplementary Materials

The following supporting information can be downloaded at: https://www.mdpi.com/article/10.3390/children9050681/s1. Figure S1: Example of Treg gate strategy partially derived from the manufacturer’s protocol (modified from https://www.bc-cytometry.com/PDF/DataSheet/B53346DS.pdf accessed on 18 January 2022). Figure S2: Example of B cell subsets gate strategy derived from the manufacturer’s protocol (modified from https://www.bc-cytometry.com/PDF/DataSheet/B53318DS.pdf accessed on 18 January 2022). Figure S3: Disease severity of children with CoV2 infection, according to age. Figure S4: Serum cytokine levels. IL10, IL12p70, IL2, and IL4 levels were measured in sera samples of patients at data entry, using ELLA Assay (Biotechne, both from R&D Systems).
