# Supplementary material for: Immunopathology of SARS-CoV-2 Infection: A Focus on T Regulatory and B Cell Responses in Children Compared with Adults
Source: Children (Basel). 2022 May 7;9(5):681. doi: 10.3390/children9050681 (PMC9139466; doi:10.3390/children9050681)
Supplement: Supplementary file 1 [file children-09-00681-s001.zip › children-1681123-supplementary.pdf]

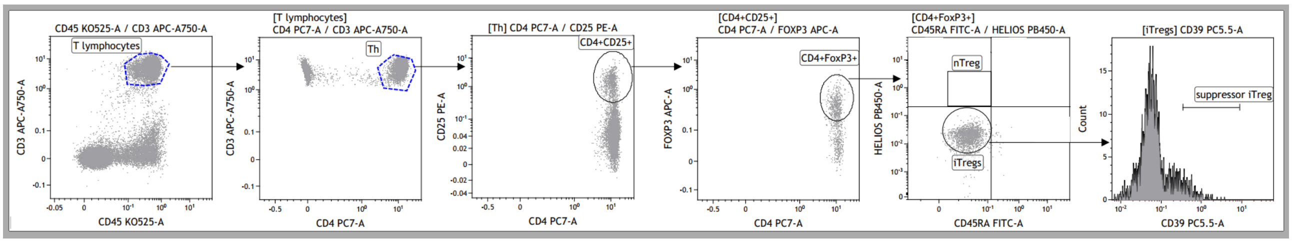

Figure S1. Example of Treg gate strategy partially derived from manufacturer's protocol.

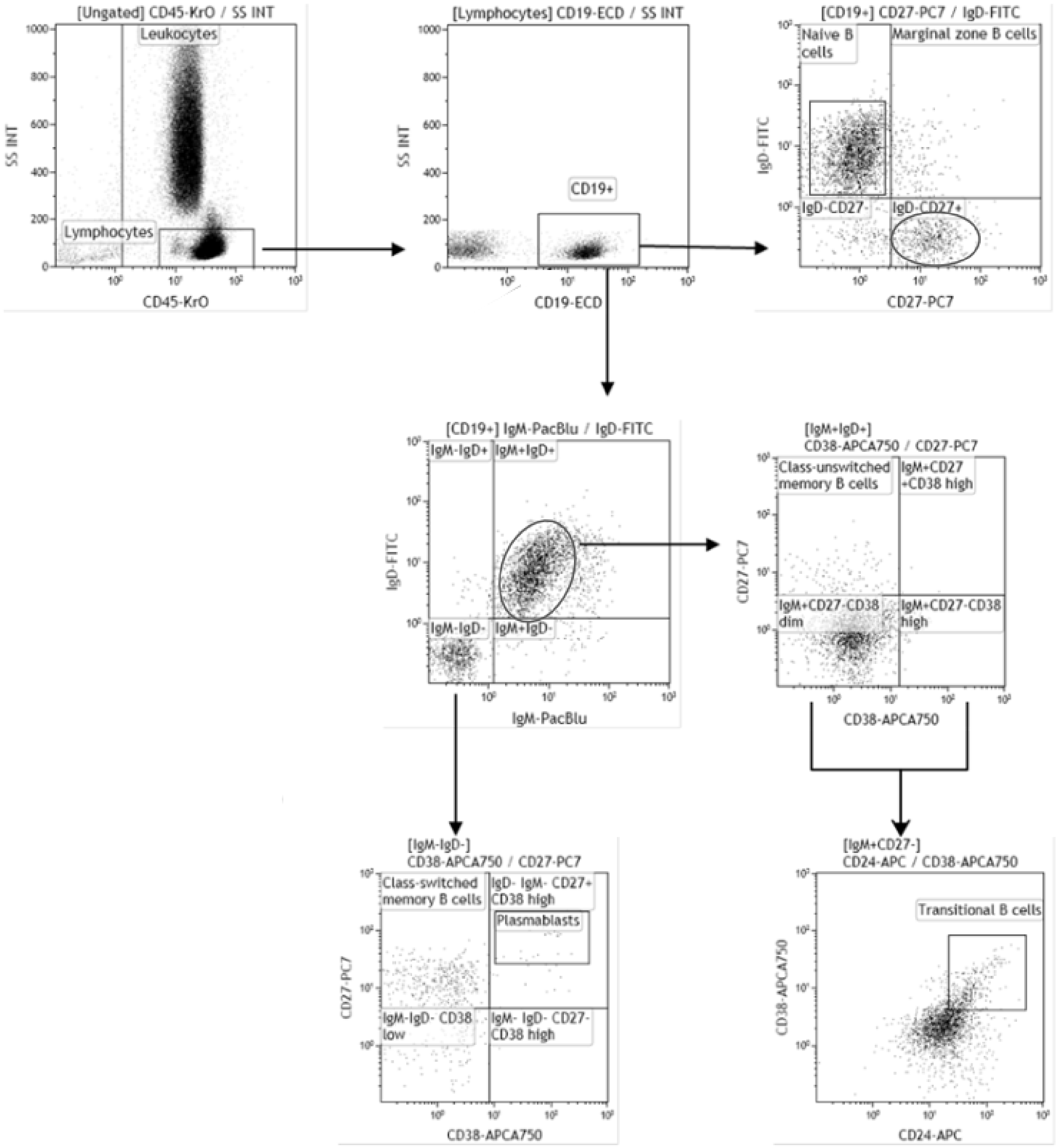

Figure S2. Example of B cell subsets gate strategy derived from manufacturer's protocol.

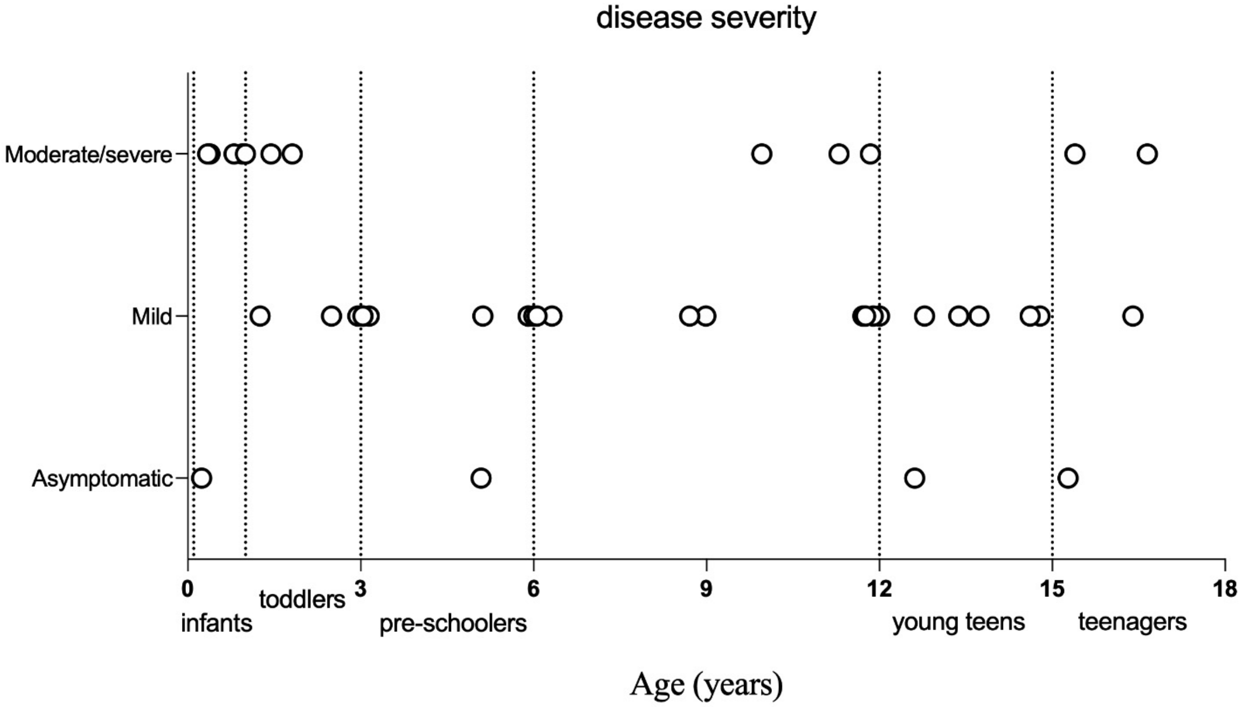

Figure S3. Disease severity of children with CoV2 infection, according to age.

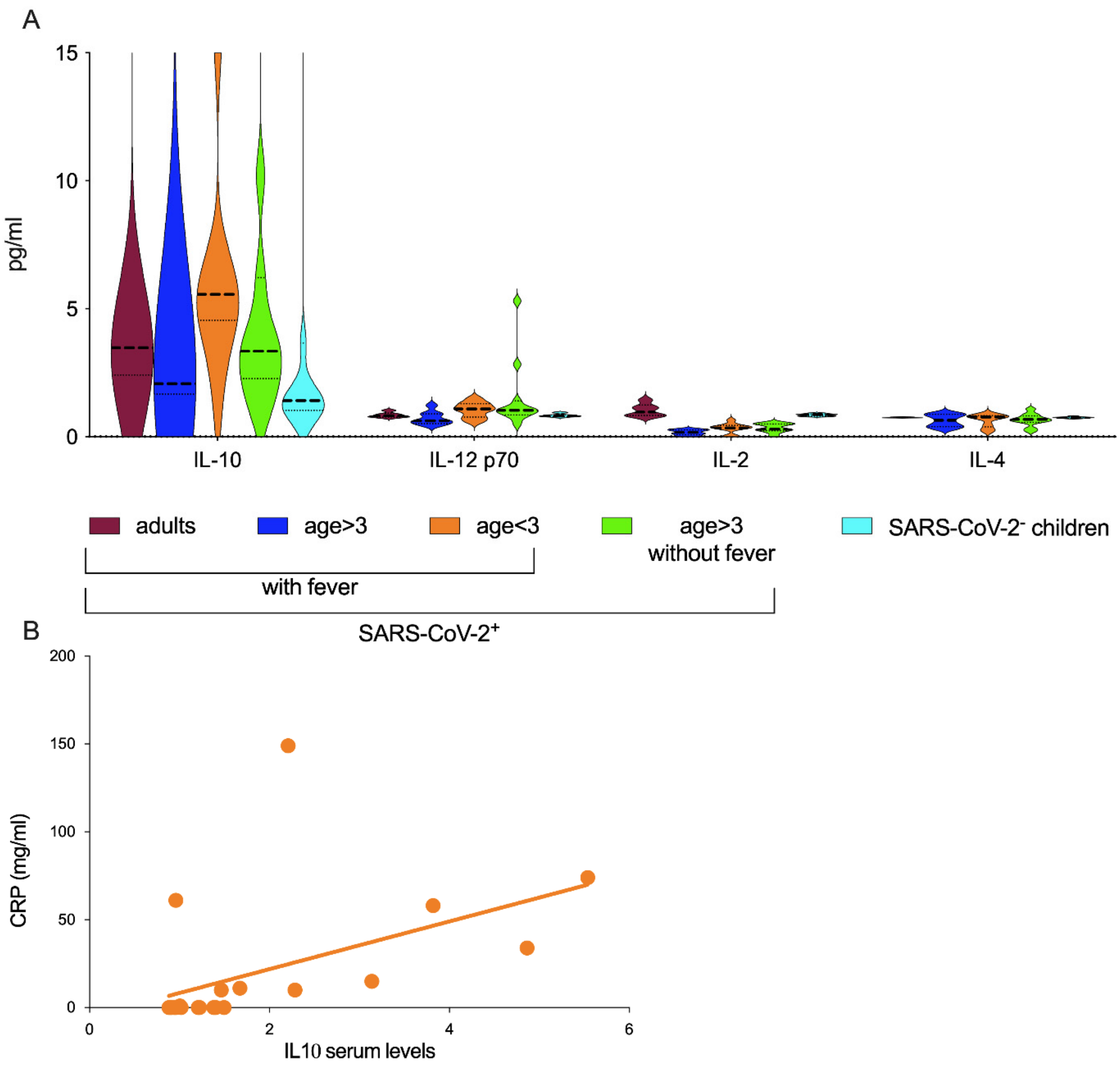

Figure S4. Serum cytokine levels. IL10, IL12p70, IL2 and IL4 levels were measured in sera samples of patients at data entry, using ELLA Assay (**A**). (**B**) shows correlation between C-Reactive Protein and Interleukin 10 levels.
